# Effects of Dietary Phosphorus Level on the Expression of Calcium and Phosphorus Transporters in Laying Hens

**DOI:** 10.3389/fphys.2018.00627

**Published:** 2018-05-25

**Authors:** Peng Li, Rongmei Wang, Hongchao Jiao, Xiaojuan Wang, Jingpeng Zhao, Hai Lin

**Affiliations:** ^1^Shandong Provincial Key Laboratory of Animal Biotechnology and Disease Control and Prevention, College of Animal Science and Veterinary Medicine, Shandong Agricultural University, Tai’an, China; ^2^College of Sports Medicine and Rehabilitation, Taishan Medical University, Tai’an, China

**Keywords:** dietary phosphorus, phosphorus transporter, calcium transporter, eggshell, laying hens

## Abstract

The transport of calcium and phosphorus is mainly relied on their corresponding transporters. The aim of this study was to determine the effect of dietary phosphorus level on the expression of the relevant calcium and phosphorus transporters in laying hens, which has a large amount of calcium and phosphorus input from intestine and output from kidney and eggshell gland. Thirty-six 25-week-old Hy-line Brown hens were fed diets with different available phosphorus level (AP, 0.15, 0.41, and 0.82%), respectively. The expression of phosphorus transporters type IIa and type IIb Na/Pi co-transporter (NPt2a, NPt2b), calcium transporter calbindin-D28k (CaBP-D28k), and plasma membrane Ca ATPase 1b (PMCA1b) were measured in small intestine, kidney, and eggshell gland by RT-PCR and western blot. The results showed that serum calcitriol and PTH concentrations were not affected (*P* > 0.05) by dietary AP levels. Duodenum had the highest mRNA and protein expression level of NPt2b than jejunum and ileum (*P* < 0.05). The protein expression abundance of CaBP-D28k and PMCA1b were higher in duodenum than that in jejunum and ileum (*P* < 0.05). 0.15%-AP diet upregulated the ileal mRNA expression level of NPt2b and renal mRNA expression level of NPt2a (*P* < 0.05), while downregulated the protein abundance of NPt2b and CaBP-D28k mRNA expression in shell gland (*P* < 0.05). In conclusion, both the Ca and P transporters were highly expressed in duodenum. Low AP diet decreased protein expression abundance of NPt2b in duodenum while upregulated the mRNA expression level of NPt2a in kidney. The result suggests that both the phosphorus absorption in proximal intestine and its reabsorption in kidney are involved in the adaption to low AP diet.

## Introduction

Calcium (Ca) and phosphorus (P) are essential nutrients that play a critical role in many biological processes. They are important for bone development and mineralization in the form of hydroxyapatite (99% Ca, 80% P) (Veum et al., 2010). The balance of Ca and P is maintained by the absorption in small intestine, reabsorption and excretion of kidney, and deposition and mobilization of bone. In laying hens, Ca and P are involved in the formation of eggshell during laying period. The chemical composition of the chicken eggshell (by weight) is calcium carbonate (94%), magnesium carbonate (1%), calcium phosphate (1%) and organic matter (4%) (Stadelman, 2000). With a large amount of Ca and P intake on the one hand, and high Ca excretion from eggshell gland on the other hand, laying hens can serve as an interesting model of Ca and P outflow, which is involved in the development of osteoporosis.

Ca and P are two important minerals for laying hens to affect productive performance and eggshell quality (Clunies et al., 1992; Schreiweis et al., 2003). The unsynchronous rise of Ca content with increased eggshell weight is involved in the deteriorated eggshell quality as laying hen ages (Bustany and Elwinger, 1987; Bolukbasi et al., 2005). Phosphorus affects the Ca deposit during bone formation, and thereby affecting the Ca content in eggshell (Ahmad and Balander, 2003). Phosphorus plays an important role in eggshell formation and metabolism, in spite of there is little phosphorus in eggshell (Said et al., 1984; Rao et al., 1992). Increasing plasma P can result in decreased egg specific gravity (Miles and Harms, 1982).

Two Na/Pi cotransporter families, Na/Pi-II and NaPi-III, show conserved structural features. In vertebrates, the slowly adapting intestinal P absorption as well as the acutely regulated renal P reabsorption is both mediated by NaPi-II related proteins (Murer and Biber, 1996; Hilfiker et al., 1998), which locates at the apical sites of epithelial cells in small intestine and kidney (Murer et al., 2004). In small intestine, P absorption is primary adjusted by type IIb Na/Pi cotransporter (NPt2b) (Werner and Kinne, 2001; Marks et al., 2006; Yan et al., 2007; Sabbagh et al., 2009), whereas renal P reabsorption is mediated by type IIa and IIc Na/Pi cotransporter (NPt2a, NPt2c) (Werner and Kinne, 2001; Murer et al., 2004; Biber et al., 2009). The NPt2b cotransporter is the major transporter in small intestine that primary expressed at the brush-border membranes of epithelium (Hilfiker et al., 1998). In kidney, NPt2a, as an electrogenic transporter, is mainly expressed in the brush border membrane (BBM) and has a priority to combine with phosphorus, while NPt2c is electroneutral transporter and has lower combination with phosphorus (Custer et al., 1994; Manghat et al., 2014). In chickens, the NPt2a is expressed in kidney (Werner and Kinne, 2001). The expression of NPt2a changes with plasma phosphorus in laying hens (Huber et al., 2006).

There are four models for Ca absorption in small intestine: facilitated diffusion, vesicular trafficking, transcaltachia, and regulated paracellular transport. The cytoplasmic Ca binding protein calbindin D is the central player in the facilitated diffusion model (Fleet and Schoch, 2010). Calbindin D9k (CaBP-D9k) and D28k (CaBP-D28k) are major Ca transcellular diffusion transporters in small intestine and kidney of mammals (Proszkowiec-Weglarz and Angel, 2013), which bind Ca and move it from the BBM to the basolateral membrane (Gross and Kumar, 1990; Nemere et al., 1991). Unlike mammals, CaBP-D28k is the primary Ca transcellular diffusion transporters in small intestine of birds (Wasserman and Taylor, 1966). The final step of facilitated diffusion is extruding Ca from cells (Fleet and Schoch, 2010), which is mainly achieved by the plasma membrane Ca ATPase (PMCA), moving Ca across the basolateral membrane to the outside of the cell (Carafoli, 1991). The plasma membrane Ca ATPase 1b (PMCA1b) is the primary isomer expressed in small intestine and kidney of chicken (Melancon and De Luca, 1970; Davis et al., 1987; Qin and Klandorf, 1993; Quinn et al., 2007).

The Na/Pi cotransporters in small intestine and kidney are regulated by dietary P (Segawa et al., 2002, 2005). In chickens, dietary P deficiency leads to the upregulation of NPt2b and CaBP-D28k in the small intestine and NPt2a in the kidney (Proszkowiec-Weglarz and Angel, 2013). Low P and low Ca diets upregulate the mRNA expression level of PMCA (Hurwitz et al., 1987). In laying hens, however, the effect of dietary P on the expression level of Ca and P transporters CaBP-D28k, PMCA1b, NPt2b, and Npt2a remains to be elucidated. Hence, we hypothesized that dietary P level would have an effect on the expression of both Ca and P transporter in small intestine, kidney, and eggshell gland.

In this study, 25-weeks-old Hy-line laying hens were employed as the experimental animals. The aim of the present study was to determine whether dietary phosphorus level affects the expression of P and Ca transporters in small intestine, kidney, and shell gland in laying hens.

## Materials and Methods

All procedures used in this study were approved by the Animal Care Committee of Shandong Agricultural University (China) and were carried out in accordance with the guidelines for experimental animals of the Ministry of Science and Technology (Beijing, China).

### Animals and Diets

A total of 36 Hy-line Brown laying hens (25 weeks of age) with similar body weight (1.80 ± 0.10 kg) and laying performance (88 ± 1.10% laying rate) were obtained from a local company (Qingdao Aote Laying Hen Breeding Farm, China). The hens were randomly divided into three groups of 12 hens reared in cage with one per cage. The three groups of hens were fed with one of the three experimental diets: basal corn-soybean meals diet with 0.41% available P (AP, Control), 0.15% AP diet (Low-P), and 0.82% AP diet (High-P) at the same time (**Table 1**). Each cage served as one replicate. All the hens had free access to feed and water during experimental period. The experiment lasted for 2 weeks. Feed intake and egg numbers were recorded daily. The experiment was carried out in Animal Science and Technology Experimental Station, Shandong Agricultural University and performed in accordance with the “Guidelines for Experimental Animals” of the Ministry of Science and Technology (Beijing, China). The experimental protocol and zootechnical performance (feed intake and hen-day egg production) had been reported by Wang et al. (2018), which was part of the present study.

**Table 1:** Composition of experimental diets (air-dry basis).

Items	Content (%)		
	0.15% AP	0.41% AP	0.82% AP
**Ingredients**			
Corn	60.72	60.72	60.72
Bran	25.39	25.39	25.39
Limestone	9.73	8.76	7.22
CaHPO_ 4_	–	1.62	4.18
Soybean oil	1.43	1.43	1.43
Maifan stone^1^	1.70	1.05	0.03
NaCl	0.32	0.32	0.32
Lys	0.23	0.23	0.23
Met	0.14	0.14	0.14
Choline chloride	0.09	0.09	0.09
Mineral Premix^2^	0.20	0.20	0.20
Vitamin Premix^2^	0.05	0.05	0.05
Total	100.00	100.00	100.00
**Nutrient levels**			
CP	16.50	16.50	16.50
ME (MJ/Kg)	11.3	11.3	11.3
Ca^3^	3.86	3.76	3.60
Total P^3^	0.39	0.73	1.25
Available P	0.15	0.41	0.82
Lys	0.95	0.95	0.95
Met	0.41	0.41	0.41
Met + Cys	0.70	0.70	0.70

*^1^Maifan stone without calcium and phosphorus. ^2^Provided per Kg of diet: vitamin A, 8,000 IU; vitamin D_3_, 3,200 IU; vitamin E, 20 IU; vitamin K, 3 mg; thiamin 1.7 mg; riboflavin, 5.5 mg; niacin, 28 mg; pantothenic acid, 6.6 mg; pyridoxine, 3.3 mg; biotin, 0.1 mg; folic acid, 0.6 mg; vitamin B_12_, 0.022 mg; Mn,88 mg; Zn, 88 mg; Fe, 55 mg; I, 1.7 mg; Cu, 5.5 mg; Se, 0.3 mg. ^3^Measured values.*.

### Tissue Sampling and Preparation

At the end of the trial, all hens were fasted overnight, and eight hens were randomly selected from each treatment. After a blood sample was obtained from a wing vein, the hens were euthanized by cervical dislocation then dissected, and the duodenum mucosa, jejunum mucosa, ileum mucosa, kidney, and shell gland mucosa were sampled. Tissue samples were immediately snap-frozen in liquid nitrogen and stored at -80°C for further analysis. Serum was separated by centrifugation at 1,500 *g* for 15 min and stored at -20°C until analysis. The eggs were collected during the last 3 days of the experiment and used for egg quality measurement. The following parameters were analyzed: egg quality, the mRNA expression of NPt2b, NPt2a, CaBP-D28k, PMCA1b, and the protein expression of NPt2b, CaBP-D28k, PMCA1b.

### Serum Hormone Analysis

Serum calcitriol and parathyroid hormone (PTH) were measured with radioimmunoassay by using a donkey anti-sheep and sheep anti-human serum, respectively, (Beijing North Institute of Biological Technology, Beijing, China). The hormones measured in this study were referred to as immunoreactive hormones. The sensitivities of the assays for calcitriol and PTH were 20 pg/mL and 10 ng/dL, respectively. All of the serum samples were included in the same assay to avoid interassay variability. The intraassay coefficients of variations were 9.7, and 6% for calcitriol and PTH, respectively.

### Egg Quality Measurement

Egg shape index was calculated by long diameter/short diameter. After breakout, yolk was separated and weighed. Relative weight of yolk was calculated against egg weight. The albumen height, Haugh unit were detected by the multifunctional egg detector (EMT-5200, Japan Robotmation). Eggshell breaking strength was determined based upon the vertical axis measured by an eggshell strength tester (EFG-0503, ROBOTMATION, Japan). Shell thickness was determined at the sharp, blunt ends, and equator by an eggshell strength tester (EFG-0503, ROBOTMATION, Japan), and took the average of the three data. Eggshells were weighed after dried.

### Real-Time PCR Analyses

Total RNA was extracted from intestine, kidney, and eggshell gland using TransZol Up (TransGen Biotech, China). Then the concentration of the RNA was measured by spectrophotometry (Eppendorf, Germany), and verified the RNA purity by calculating the ratio between the absorbance values at 260 and 280 nm (A260/280 ≈ 1.75–2.01). Next, reverse transcription was performed using total RNA (1 μg) for first-strand cDNA synthesis with the Transcriptor First Strand cDNA Synthesis Kit (Roche, Germany). The cDNA was amplified in a 20 μl PCR reaction system containing 0.2 μmol/L of each specific primer (Sangon, China) and of the SYBR Green master mix (Roche, Germany) according to the manufacturer’s instructions. Real-time PCR was performed at the ABI QuantStudio 5 PCR machine (Applied Biosystems; Thermo, United States), the primers were as follows: chicken NPt2b: forward, ACTGGCTTGCTGTGTTTGC and reverse, AGGGGCATCTTCACCACTTT; NPt2a: forward, CCAAACTGCACGGCTTCT and reverse, TGGGAGGTCAGT GTTGATGA; CaBP-D28k: forward, TGTTATGGAGTGCAGG ATGG and reverse, TAGAGCGAACAAGCAGGTGA; PMCA1b: forward, TTCAGGTACTCATGTGATGGAAGG and reverse, CAGCCCCAAGCAAGGTAAAG; β-actin: forward, CTGGC ACCTAGCACAATGAA and reverse, CTGCTTGCTGATCCA CATCT. Primers against β-actin was used as internal controls, and all of the mRNA values were normalized the differences between individual samples.

### Western Blot Analyses

The tissue samples were homogenized in 1 mL of lysis buffer (Beyotime, China). After centrifuged at 12000 *g* for 10 min at 4°C, the supernatant was collected and then quantified for protein by the method of BCA protein assay kit (Beyotime, China) according to the manufacturer’s protocol. An equal amount of proteins (18 μg) were separated by 7.5–10% SDS polyacrylamide gels (Bio-Rad, Richmond, 246 CA) and the proteins were transferred onto polyvinylidene fluoride membrane (Millipore, United States) at 200 mA for 2 h in a Tris-glycine buffer with 20% anhydrous ethanol at 4°C. Then membranes were blocked with western blocking buffer (Beyotime, China) for 1 h at room temperature. The membranes were incubated with specific primary antibodies at 4°C with gentle shaking overnight. The primary antibodies used were anti-NPt2b (GenScript, China), anti-Calbindin-d28k (Sigma, United States), anti-PMCA (Thermo, United States) and anti-β-actin (Beyotime, China). The membrane was washed with Tris-buffered saline/Tween buffer for 10 min three times, and then the membranes were incubated with secondary antibodies (HRP-conjugated anti-rabbit or anti-mouse IgG, 1:1000; Beyotime) for 4 h at 4°C. After being washed as before, membranes were then visualized by exposure to Hyperfilm ECL (Beyotime, China). Western blots were developed and quantified using BioSpectrum 810 with VisionWorks LS 7.1 software (UVP LLC). The band intensity was normalized to the β-actin band in the same sample.

### Statistical Analyses

Data were presented as the means ± SEM (*n* = 8 or 12). All the data were analyzed with one-way ANOVA by using the STATISTICAL ANALYSIS Software (version 8e; SAS Institute, Cary, NC, United States) to estimate the main effect of dietary AP level. When the main effect of the treatment was significant, the differences between means were assessed by Tukey’s honestly significant difference test. *P* < 0.05 was considered statistically significant.

## Results

### Effects of Dietary Available Phosphorus Level on Serum Hormones and Egg Quality

Dietary AP level had no significant influence on egg weight, egg shape index, shell thickness, shell strength, albumen height, Haugh units, percentage of shell, and percentage of yolk (*P* > 0.05, **Table 2**). Dietary AP level had no significant influence on serum calcitriol and PTH (*P* > 0.05, **Table 3**). Feed intake and egg production were not changed by dietary AP treatment and reported elsewhere (Wang et al., 2018).

**Table 2:** Effects of dietary available phosphorus level on egg quality of laying hens.

Items	0.15% AP	0.41% AP	0.82% AP	*P*
Egg weight, g	58.55 ± 1.01	58.52 ± 0.99	58.79 ± 1.45	0.984
Egg shape index	1.27 ± 0.01	1.29 ± 0.01	1.27 ± 0.01	0.488
Shell thickness, mm	0.32 ± 0.01	0.33 ± 0.002	0.34 ± 0.01	0.253
Shell hardness, kg⋅f	3.89 ± 0.20	4.00 ± 0.19	4.11 ± 0.07	0.671
Albumen height, mm	7.55 ± 0.18	7.33 ± 0.21	7.01 ± 0.23	0.209
Haugh units, HU	87.08 ± 1.15	85.03 ± 1.43	83.70 ± 1.34	0.220
Percentage of shell, %	10.56 ± 0.22	10.82 ± 0.16	10.88 ± 0.20	0.486
Percentage of yolk, %	24.23 ± 0.23	24.17 ± 0.43	24.18 ± 0.20	0.988

*Data were presented as means ± SEM (n = 12)*.

**Table 3:** Effects of available phosphorus level on serum calcitriol and parathyroid hormone (PTH) of laying hens.

Serum	0.15% AP	0.41% AP	0.82% AP	*P*
Calcitriol, pg/mL	24.82 ± 1.47	32.64 ± 2.96	28.08 ± 3.78	0.183
PTH, ng/dL	25.59 ± 3.14	33.21 ± 3.28	30.92 ± 2.07	0.184

*Data were presented as means ± SEM (n = 8)*.

### The Expression of NPt2b, CaBP-28K, and PMCA1b in Small Intestine of Hens Feeding Normal Diet

**FIGURE 1:**
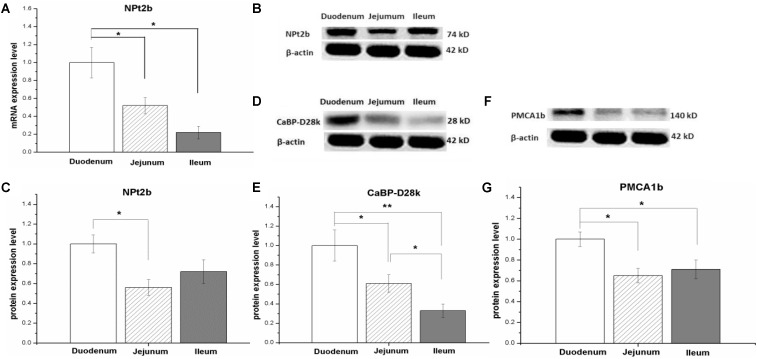
The mRNA and protein expression level of calcium and phosphorus transporters in small intestinal tract of laying hens fed with control diet (0.41% AP). The type II-B Na/Pi and co-transporter (NPt2b) in small intestine **(A–C)**; the protein expression level of Calbindin-D28k (CaBP-D28k) **(D,E)**; the protein expression level of plasma membrane calcium ATPase 1b (PMCA1b) **(F,G)**. Data were presented as means ± SEM (*n* = 8); ^∗^*P* < 0.05, ^∗∗^*P* < 0.01.

In control hens, the mRNA expression level of NPt2b was the highest in the duodenum, followed by the jejunum and ileum (*P* < 0.05, **Figure 1A**). Similarly, the protein expression abundance of NPt2b in the small intestine was the highest in the duodenum and the lowest in the jejunum (*P* < 0.05, **Figures 1B,C** and Supplementary Figure S1). The protein expression abundance of CaBP-28K and PMCA1b in the small intestine was relative higher in duodenum than that in jejunum and ileum (*P* < 0.05, **Figures 1D–G** and Supplementary Figures S2, S3).

### Effects of Dietary Available Phosphorus Level on Calcium and Phosphorus Transporter Expression in Small Intestine

**FIGURE 2:**
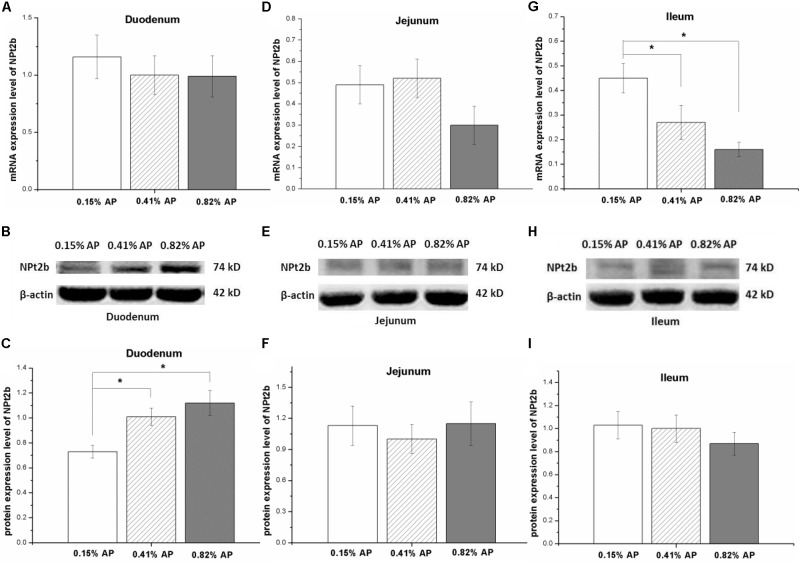
Effect of dietary available phosphorus levels (AP, 0.15, 0.41, and 0.82%) on the mRNA and protein expression level of type II-B Na/Pi co-transporter (NPt2b) in small intestine of laying hens. Duodenum **(A–C)**; jejunum **(D–F)**; ileum **(G–I)**. Data were presented as means ± SEM (n = 8); ^∗^*P* < 0.05.

**FIGURE 3:**
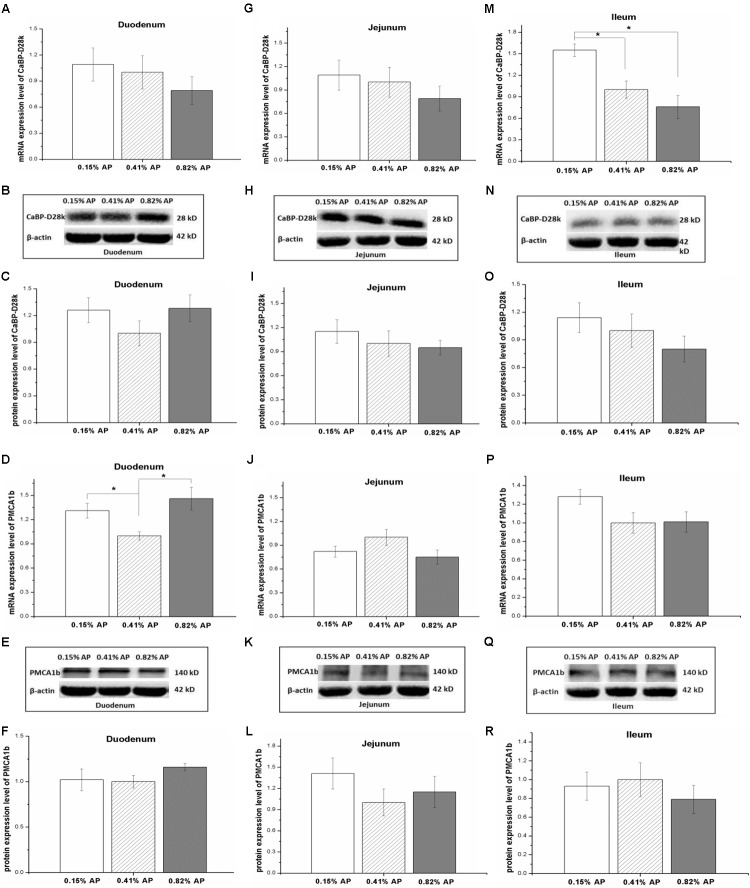
Effects of dietary available phosphorus levels (AP, 0.15, 0.41, and 0.82%) on mRNA and protein expression levels of Calbindin-D28k (CaBP-D28k) and plasma membrane calcium ATPase 1b (PMCA1b) in small intestine of laying hens. Duodenum **(A–F)**; jejunum **(G–L)**; ileum **(M–R)**. Data were presented as means ± SEM (*n* = 8); ^∗^*P* < 0.05.

The mRNA expression level of NPt2b in the duodenum and jejunum was not affected by dietary phosphorus level (*P* > 0.05, **Figures 2A,D**). However, the mRNA expression level of NPt2b in ileum was significantly upregulated in 0.15% AP group, compared with both 0.41 and 0.82% AP groups (*P* < 0.05, **Figure 2G**). The ileal mRNA expression level of CaBP-D28k was upregulated by 0.15% AP diet (*P* < 0.05, **Figure 3M**), and there were no difference in duodenum and jejunum (*P* > 0.05, **Figures 3A,G**). The duodenal mRNA expression level of PMCA1b was upregulated by 0.15% and 0.82% AP diet (*P* < 0.05, **Figure 3D**), while there were no difference in jejunum and ileum (*P* > 0.05, **Figures 3J,P**).

The protein expression abundance of NPt2b in duodenum was significantly lower in 0.15% AP than that in 0.41 and 0.82% AP groups (*P* < 0.05, **Figures 2B,C** and Supplementary Figure S4). In contrast, the protein abundance of NPt2b was not altered by dietary AP level in jejunum and ileum (*P* > 0.05, **Figures 2E,F,H,I** and Supplementary Figures S5, S6). Unlike the mRNA expression, dietary phosphorus level had no significant influence on the protein expression abundance of CaBP-D28k and PMCA1b in small intestine (*P* > 0.05, **Figures 3B–R** and Supplementary Figures S7–S12).

### Effects of Dietary Available Phosphorus Level on Calcium and Phosphorus Transporter Expression in Kidney

**FIGURE 4:**
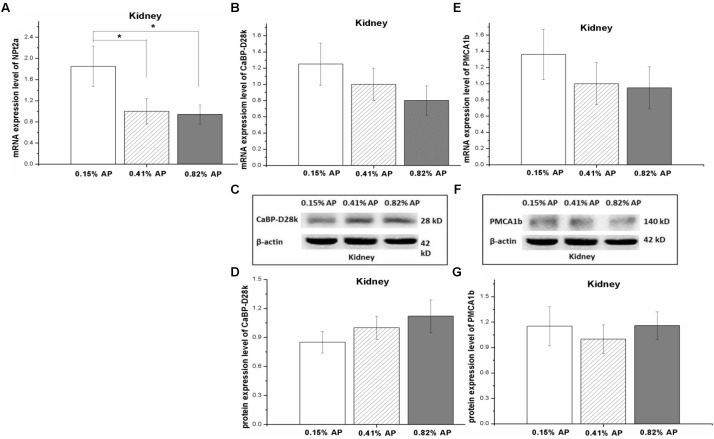
Effect of dietary available phosphorus levels (AP, 0.15, 0.41, and 0.82%) on calcium and phosphorus transporter expression in kidney of laying hens. The mRNA expression level of type II-A Na/Pi co-transporter (NPt2a) **(A)**; the mRNA and protein expression levels of Calbindin-D28k (CaBP-D28k) **(B–D)**; the mRNA and protein expression levels of plasma membrane calcium ATPase 1b (PMCA1b) **(E–G)**. The values are presented as the means ± SEM (*n* = 8); ^∗^*P* < 0.05.

The renal mRNA expression level of NPt2a was significantly increased by 0.15% AP diet treatment (*P* < 0.05, **Figure 4A**) compared with 0.41 and 0.82% AP groups. In contrast, there was no significant change in mRNA expression level of CaBP-D28k and PMCA1b (*P* > 0.05, **Figures 4B,E**). The protein expression abundance of CaBP-D28k and PMCA1b were not altered by dietary AP level (*P* > 0.05, **Figures 4C,D,F,G** and Supplementary Figures S13, S14).

### Effects of Dietary Available Phosphorus Level on Calcium Transporters Expression in Shell Gland

**FIGURE 5:**
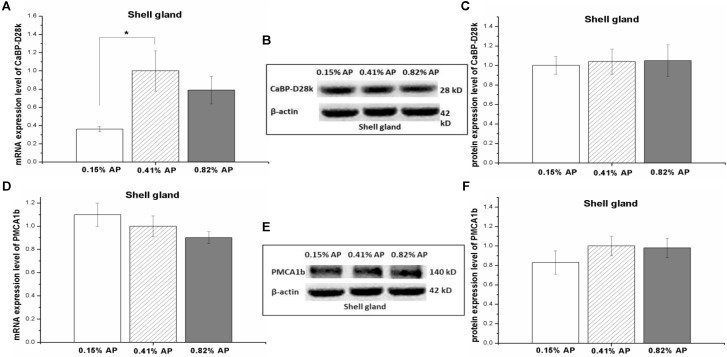
Effect of dietary available phosphorus levels (AP, 0.15, 0.41, and 0.82%) on the mRNA expression level and protein expression level of calbindin-D28k (CaBP-D28k) and plasma membrane calcium ATPase 1b (PMCA1b) in shell gland of laying hens. The mRNA and protein levels of CaBP-D28k **(A–C)**; the mRNA and protein expression levels of PMCA1b **(D–F)**. The values are presented as the means ± SEM (*n* = 8); ^∗^*P* < 0.05.

The mRNA expression level of CaBP-D28k in shell gland was significantly decreased by 0.15% AP, compared with 0.41 and 0.82% AP (*P* < 0.05, **Figure 5A**). Dietary AP level had no significant influence on mRNA expression level of PMCA1b (*P* > 0.05, **Figure 5D**). Moreover, there was no significant effect of dietary phosphorus level on protein expression abundance of CaBP-D28k and PMCA1b in shell gland (*P* > 0.05, **Figures 5B,C,E,F** and Supplementary Figures S15, S16).

## Discussion

Both the intestinal absorption and renal reabsorption contributes to the phosphorus homeostasis in chickens. In the present study, we measured the expression of Ca and P cotransporters in small intestine, kidney, and eggshell gland. The result indicated that duodenum expresses the highest level of NPt2b, CaBP-D28k, and PMCA1b, compared to jejunum and ileum. High AP diet (0.82%) upregulated the protein expression level of NPt2b in duodenum but not in jejunum and ileum. In kidney, the expression level of NPt2a was upregulated by low AP diet (0.15%). The result suggests that the dietary P level has a minor regulating effect on the protein expression abundance of calcium transporters in small intestine, kidney, and shell gland of laying hens.

### Dietary Phosphorus Level Changed Serum Phosphorus Level Without Alter Eggshell Quality

Previous studies have proved that short-term dietary treatment may change the phosphorus metabolism. In mice fed with a low-phosphorus diet for 5-days, the expression of intestinal phosphorus transport is changed (Capuano et al., 2005). In chickens, the expression of intestinal phosphorus transporter was changed by dietary phosphorus treatment within a 10-days period (Huber et al., 2015) or by a 7-days low-protein diet treatment (Xue et al., 2016). In the present study, the serum phosphorus was measured and reported elsewhere (Wang et al., 2018), which showed that serum phosphorus level was increased in 0.82% AP group while decreased in 0.15% AP group, compared to 0.41% AP hens, indicating that the experimental model was successfully built up. As feed intake was not significantly changed by dietary AP levels (reported by Wang et al., 2018), the observed response should be the result of altered dietary AP levels.

Many studies have been conducted to investigate the effects of dietary Ca and P levels on laying performance and eggshell quality. Lack of Ca and P or improper Ca/P ratio reduces eggshell quality, egg size, and egg production (Bar and Hurwitz, 1984; Härtel, 1990; Rao et al., 2006). In the present study, dietary AP level had no significant influence on eggshell quality, which was in line with the previous work by Ahmad and Balander (2003), who reported that reduction of AP from 0.45 to 0.32% had no influence on eggshell quality (egg specific gravity and shell thickness) or production performance (egg production and egg weight). As the serum calcitriol and PTH concentrations were not significantly altered by dietary AP level, the result implies that the short-term fluctuations of circulating P had no detectable harmful influence on eggshell quality.

### The Relative Expression Abundance of Calcium and Phosphorus Transporters in Small Intestine

In the present study, we evaluated the mRNA and protein expression level of Ca and P transporters in small intestine of laying hens. Duodenum expressed relative higher level of NPt2b mRNA compared to jejunum and ileum, in line with the result in broilers (Yan et al., 2007). The result indicated that the expression profile of NPt2b along with small intestinal tract was not altered by genotype of chickens. We further measured the protein abundance of NPt2b along small intestine. In accordance with the mRNA expression, duodenum had the highest protein abundance. In an *in vitro* study with ligated intestinal loops, it is proved that duodenum is the main site of P absorption, and that P absorption may be a saturated carrier mediated process in the duodenum but a non-saturated diffusion process in the jejunum or ileum of broilers (Liu et al., 2016). In ligated duodenal loops, the increased P absorption is associated with the enhanced expression level of NPt2b (Liao et al., 2017). Taken together, the result suggests that duodenum plays an important role in P absorption.

In laying hens, CaBP-D28k localizes at the intestinal enterocyte cytoplasm, and highly expresses in duodenum and followed by jejunum and ileum (Ebeid et al., 2012). In adult horses, calbindin-D9K and PMCA1/4 were highly expressed in duodenum than that in jejunum and ileum (Cehak et al., 2012). In line with the results, the present result showed that the protein expression level of CaBP-D28k and PMCA1b were higher in duodenum than that in jejunum and ileum. When the Ca intake is adequate, the absorption of Ca in different segments of small intestine is determined by the sojourn time through the intestinal tract and the solubility of Ca within the intestinal segment (Fleet and Schoch, 2010). In rats, ileum absorbs the largest amount of Ca (65%), as transit time through this segment is much longer than the proximal intestinal segments (Fleet and Schoch, 2010). Therefore, the result suggests that the expression profile of Ca and P transporters along with small intestinal tract contributes to the absorption of Ca and P at different phase in laying hens.

### Effect of Dietary Phosphorus Level on the Expression of NPt2a and NPt2b

In mouse, NPt2b is highly expressed in ileum, and low phosphorus diet stimulates the mRNA and protein expression level of NPt2b in ileum of mouse (Radanovic et al., 2005). There were similar reports to show that low phosphate diet upregulates NPt2b expression in goats and human (Huber et al., 2002; Sugiura et al., 2007). In laying hens, the mRNA level of NPt2b could be influenced by dietary P level as well. High phosphorus diets (3.43 g/kg) upregulates jejunal NPt2b mRNA expression compared to low (0.73 g/kg) and medium P diets (2.04 g/kg) (Huber et al., 2006). The duodenal mRNA level of NPt2b is increased by low-P diet while decreased by high-P diet in laying hens provided with different non-phytate-P level diets for 12 weeks (Nie et al., 2014). In line with the previous results, the present result indicated that the ileal mRNA expression level of NPt2b was upregulated by 0.15% AP diet, while the mRNA expression level NPt2b in duodenum and jejunum were not affected by dietary P level. In order to avoid the possible influence of chyme in intestinal tract on the expression of Ca and P transporters, the experimental hens were subjected to overnight feed withdrawal and were in a post-absorption state. The result suggests that the expression of NPt2b in different segments responses differently to dietary AP levels. Fed growing pigs with different phosphorus diets (0.11, 0.17, and 0.23% AP) for 3 weeks, jejunal NPt2b mRNA was upregulated by 0.11% phosphorus diets at the 1st week and then this response disappeared in the 2nd and 3rd weeks (Pokharel et al., 2017). The result implies that the changed mRNA expression level of NPt2b by low-P diet is time dependent. Hence, the time effect on NPt2b expression in laying hens needs to be investigated further.

We further measured the influence of dietary P level on protein expression abundance of NPt2b in different segments of small intestine. The decreased protein abundance of NPt2b in duodenum by 0.15% AP diet indicated that the protein expression of NPt2b in proximal intestinal segment is more sensitive to dietary AP level than that in the distal intestine segments. In this study, dietary AP level was adjusted by the supplementation of calcium hydrophosphate. The decreased NPt2b protein level in duodenum seems to be a result of reduced available phosphorus. The inconsistence expression responses in NPt2b mRNA and protein indicated that the influence of dietary AP on NPt2b expression may occur at both transcriptional and translational levels.

The adaptive capacity of kidney in P transport takes the most important role in the maintenance of P homeostasis in laying hens (Huber et al., 2006). As antibody against NPt2a is not available, the protein expression of NPt2a in kidney was not measured in the present study. In kidney, the higher NPt2a mRNA expression level in 0.15%-AP group compared to 0.41- and 0.82%-AP hens, indicated that the low phosphorus diet may increase renal P reabsorption. This result was in accordance with work of Huber et al. (2006), who reported that low-P diet (0.73 g/kg) tended to increase renal NPt2a mRNA, compared to medium (2.04 g/kg) and high P diet (3.43 g/kg). Fed pigs with diets differed in AP levels (0.23, 0.17, and 0.11%) for 3 weeks, 0.11% AP diet upregulated renal NPt2a mRNA expression (Pokharel et al., 2017). As the effect was only observed in the 2nd week, the result implies that the influence of dietary P level is time-dependent. Plasma Pi level seems to have a relevant modulatory influence on renal P reabsorption capacity in chickens (Huber et al., 2006). In this experiment, serum Pi concentration was significantly increased in 0.82% AP group while decreased in 0.15% AP chickens, compared to control group (0.41% AP, Wang et al., 2018). Hence, the regulating effect of low serum Pi on the upregulated renal NPt2a expression in 0.15%-AP hens cannot be excluded. Serum calcitriol and PTH was not significantly affected by dietary P, indicating calcitriol and PTH is not involved in the altered circulating Pi in the present experimental conditions. Moreover, signaling molecules such as fibroblast growth factor 23 is also participate in the regulation of P homeostasis (Wang et al., 2018). Hence, the effect of low phosphorus diet on the phosphorus regulating network and in turn the expression of NPt2a and NPt2b remains to be elucidated.

### Effect of Dietary Phosphorus Level on the Expression of Calcium Transporters

The expression of CaBP-D28k in intestinal tract is influenced not only by dietary Ca level but also by dietary P. In laying hens, fed different levels of non-phytate-P diets (0.20, 0.25, 0.30, 0.35, and 0.40%), the highest duodenal calbindin mRNA level is observed in 0.25% non-phytate-P diet while the highest duodenal calbindin protein abundance is detected in 0.3% non-phytate-P diet (Nie et al., 2014). The ratio of dietary P and Ca also plays an effect on the expression of calbindin in intestine. In broilers, the duodenal calbindin expression is enhanced by the imbalance between dietary Ca and non-phytate-P (Li et al., 2012). In line the previous works, the present study observed that the ileal mRNA expression level of CaBP-D28k was increased by low AP diet, and the duodenal mRNA expression level of PMCA1b was upregulated by low and high AP diets. In contrast to the mRNA expression, however, dietary AP level had no detectable influence on the protein expression of CaBP-D28k and PMCA1b in small intestine. The result suggests that the absorption of calcium is not changed by low or high AP diet in the present experimental conditions. This speculation was supported by the unchanged laying performance and eggshell quality.

We further measured the expression of CaBP-D28k and PMCA1b in kidney and eggshell gland. In horse, kidney has similar protein expression abundance of PMCA1b to duodenum (Hwang et al., 2011), indicating the important role of renal Ca reabsorption or excretion in the maintenance of Ca homeostasis. The CaBP-D28k is highly conserved in vertebrates and the localization of CaBP-28k in avian distal tubules suggests that this protein may be involved in the selective reabsorption and/or excretion of calcium in chicken as well (Rhoten et al., 1984). In our previous study (Jiang et al., 2015), the renal expression level of CaBP-D28k is not influenced by laying period (peak period, 90% laying rate vs. post-peak period, less than 80% laying rate) or by the dietary sodium bicarbonate (0.3% NaHCO_3_) supplementation. In present study, the unchanged mRNA and protein expression level of CaBP-D28k and PMCA1b in kidney indicated that the Ca transport is not influenced by dietary AP level in a relative short period.

It is reported that there is a significant correlation between the expression of CaBP in eggshell gland and the content of calcium in shell (Bar et al., 1984; Nys et al., 1986). The presence of an egg and calcium deposition in the shell gland may be a stimulatory factor for synthesis and accumulation of CaBP-D28K mRNA (Ieda et al., 1995). TRPV6 and CaBP-D28k exert important effect on delivering active calcium in shell gland and their mRNA and protein expression levels increase during eggshell formation (Yang et al., 2013). In this study, low-P diet significantly downregulated the mRNA expression level of CaBP-D28k in eggshell gland. In contrast, the protein expression abundance of CaBP-D28k and PMCA1b had no detectable difference among the three dietary P, suggesting that dietary P level has no obvious effect on calcium secretion of eggshell gland. This speculation was supported by the unchanged serum Ca (Wang et al., 2018), calcitriol and PTH concentrations, and eggshell quality.

## Conclusion

The calcium transporter CaBP-D28k and PMCA1b and phosphorus transporter NPt2b were highly expressed in duodenum than that in jejunum and ileum. Low AP diet decreased protein expression abundance of NPt2b in duodenum while upregulated the mRNA expression level of NPt2a in kidney. The protein expression abundance of CaBP-28Dk and PMCA1b in small intestine, kidney, and eggshell gland were not influenced by dietary AP levels. The result suggests that both the phosphorus absorption in proximal intestine and its reabsorption in kidney are involved in the adaption to low AP diet, which may play a role in the maintenance of egg quality.

## Author Contributions

PL, RW, and HL conceived and designed the experiments. PL and RW performed the experiments and analyzed the data. PL and HL wrote the paper. XW, JZ, and HJ provided essential reagents. All authors read and approved the final manuscript.

## Conflict of Interest Statement

The authors declare that the research was conducted in the absence of any commercial or financial relationships that could be construed as a potential conflict of interest. The reviewer SR and handling Editor declared their shared affiliation.
