# Supplementary material for: Effects of Dietary Phosphorus Level on the Expression of Calcium and Phosphorus Transporters in Laying Hens
Source: Front Physiol. 2018 May 25;9:627. doi: 10.3389/fphys.2018.00627 (PMC5992381; doi:10.3389/fphys.2018.00627)

Original images

Fig S1 The protein expression level of NPt2b in small intestine (Fig 1B)

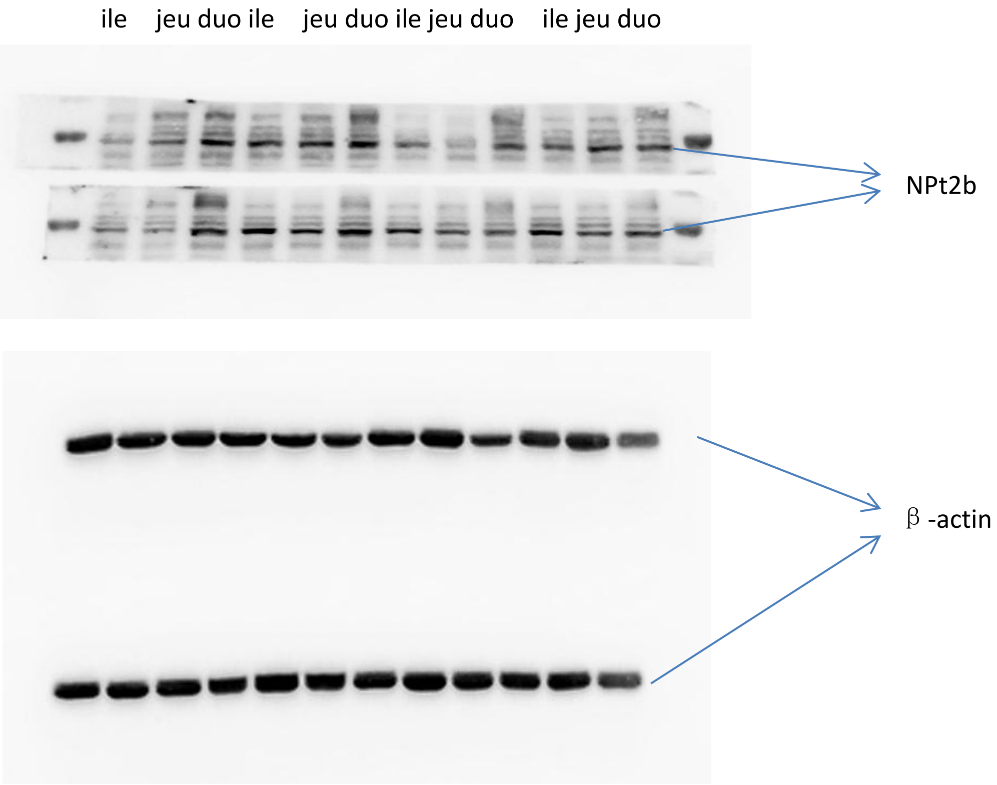

Fig S2 The protein expression level of CaBP-D28k in small intestine (Fig 1D)

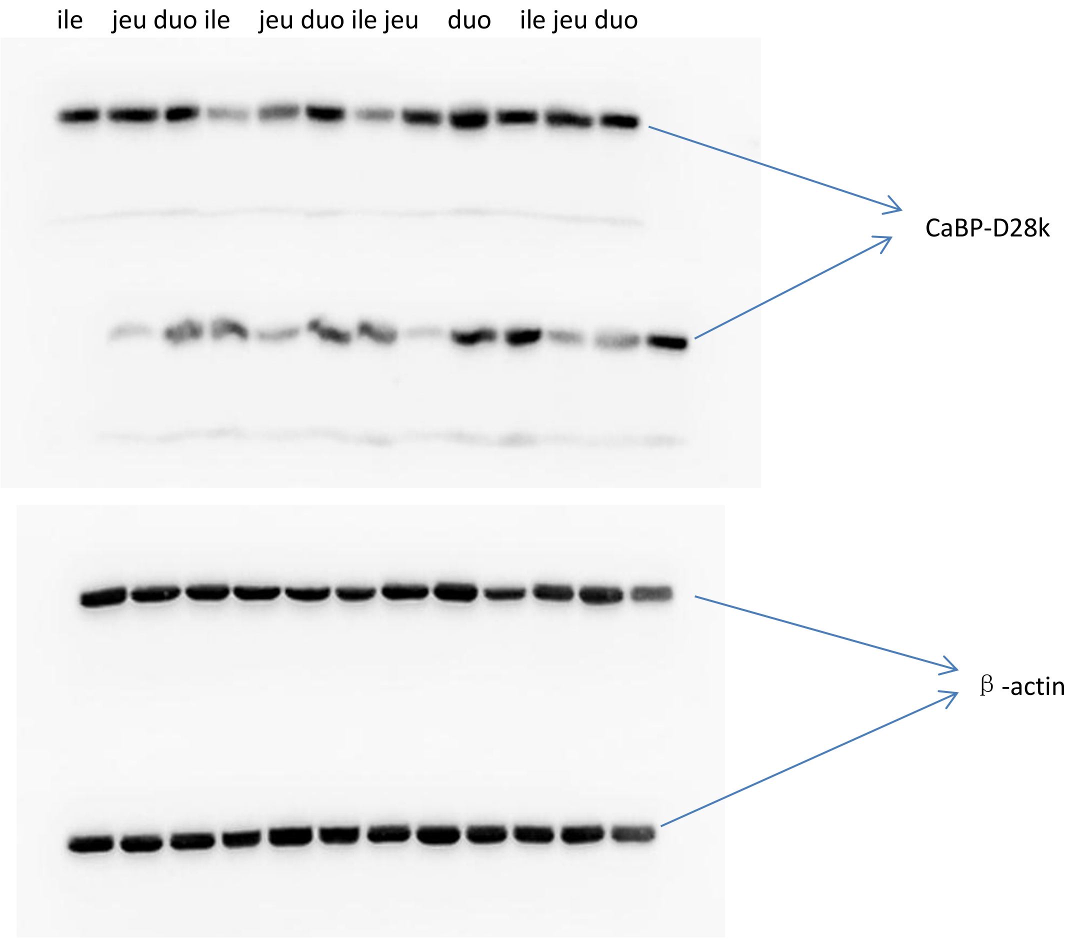

Fig S3 The protein expression level of PMCA1b in small intestine (Fig 1F)

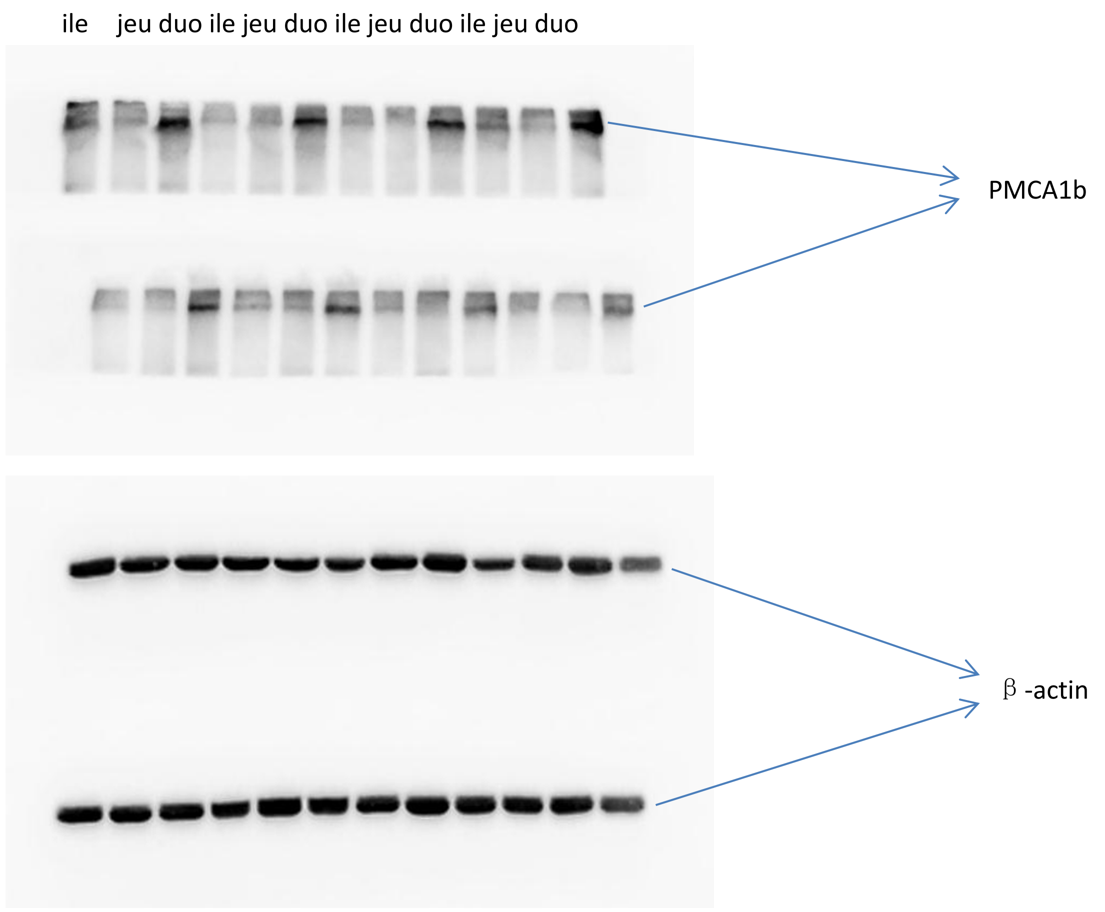

Fig S4 Effect of dietary available phosphorus levels protein expression level of NPt2b in duodenum (Fig 2B)

0.82 0.41 0.15 0.82 0.41 0.15 0.82 0.41 0.15 0.82 0.41 0.15% AP

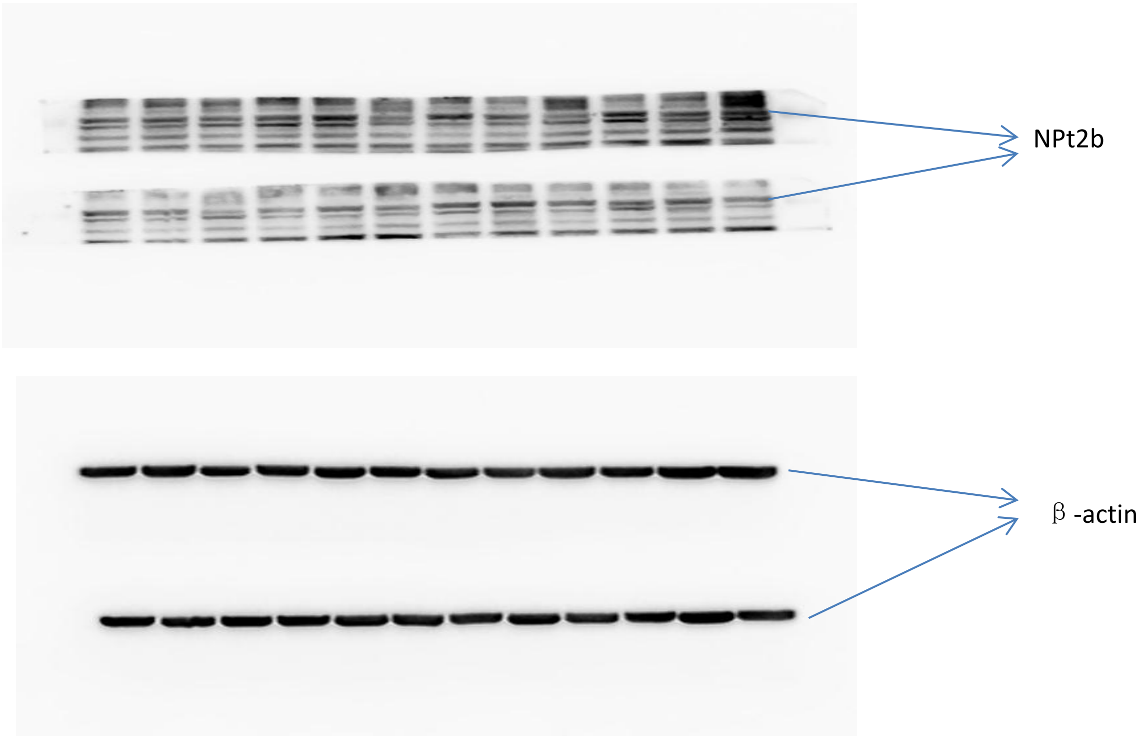

Fig S5 Effect of dietary available phosphorus levels protein expression level of NPt2b in jejunum (Fig 2E)

0.82 0.41 0.15 0.82 0.41 0.15 0.82 0.41 0.15 0.82 0.41 0.15% AP

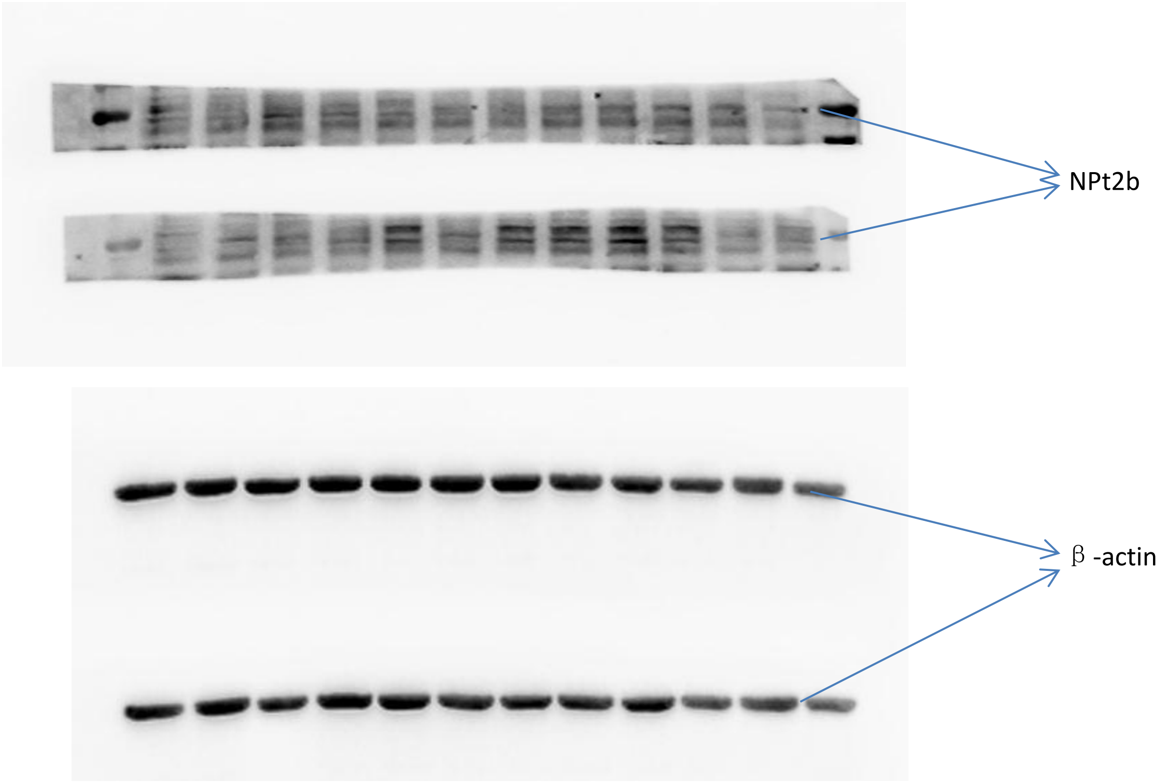

Fig S6 Effect of dietary available phosphorus levels protein expression level of NPt2b in ileum (Fig 2H)

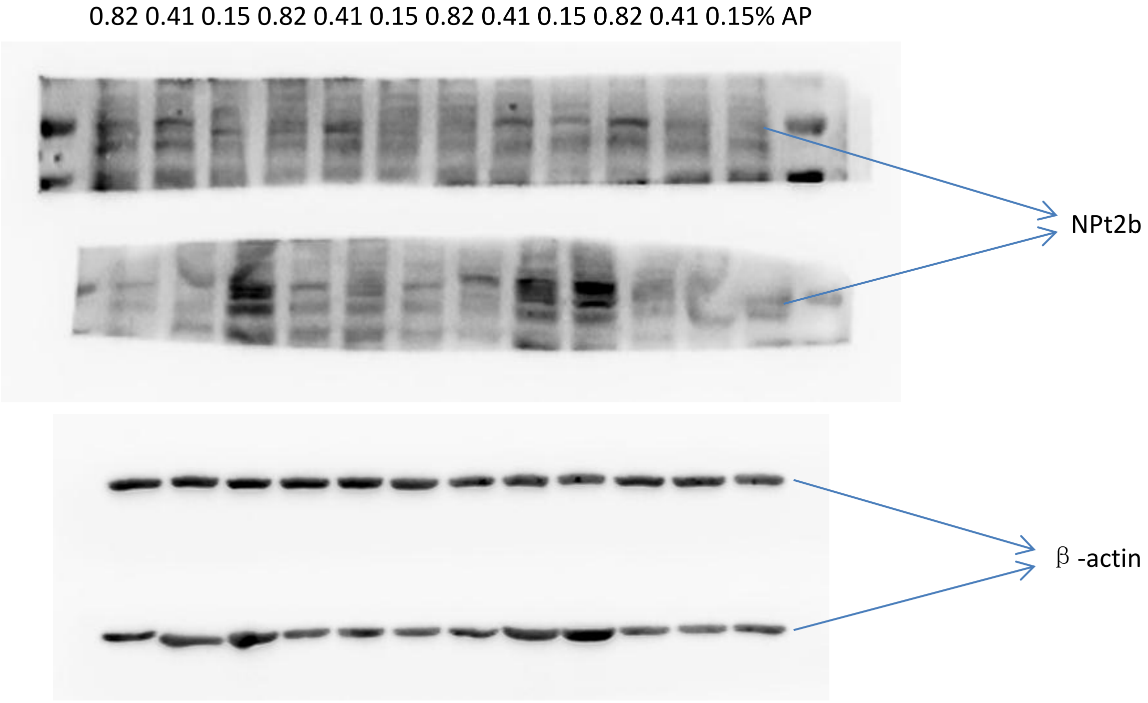

Fig S7 Effects of dietary available phosphorus levels protein expression level of CaBP-D28k in duodenum (Fig 3B)

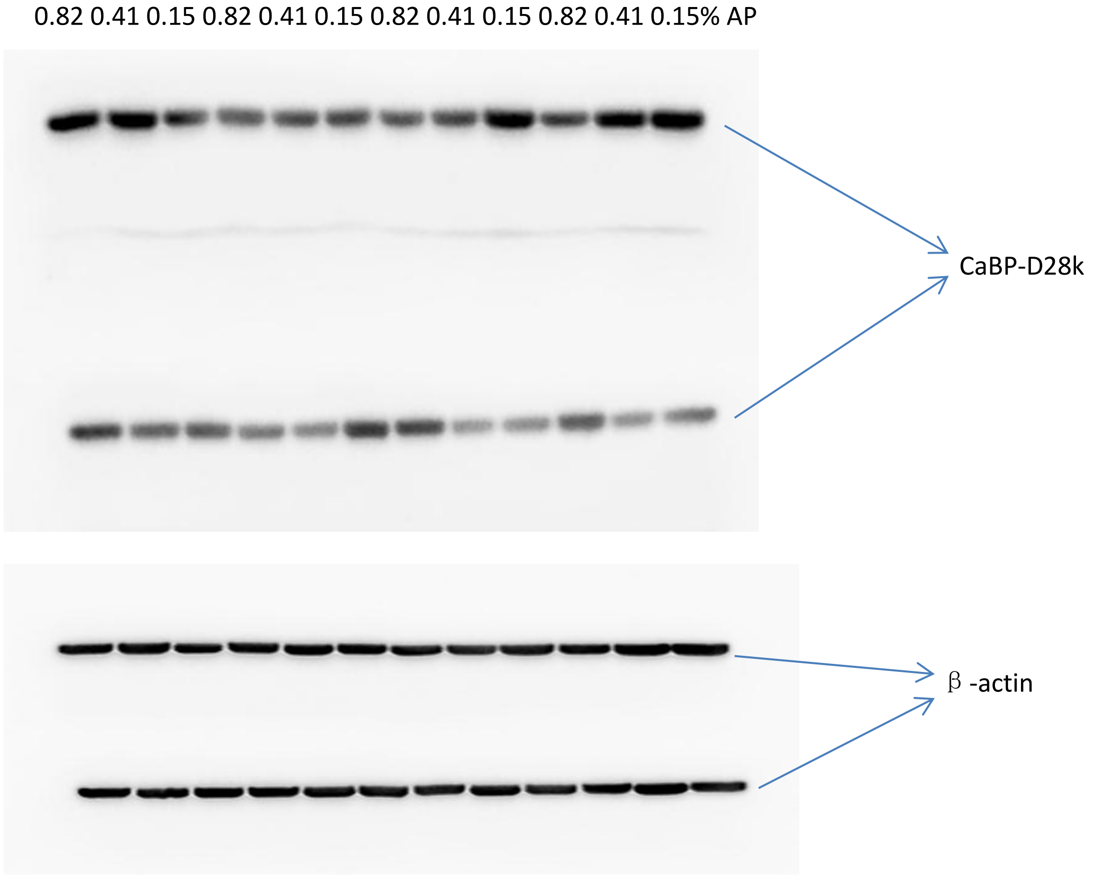

Fig S8 Effects of dietary available phosphorus levels protein expression level of PMCA1b in duodenum (Fig 3E)

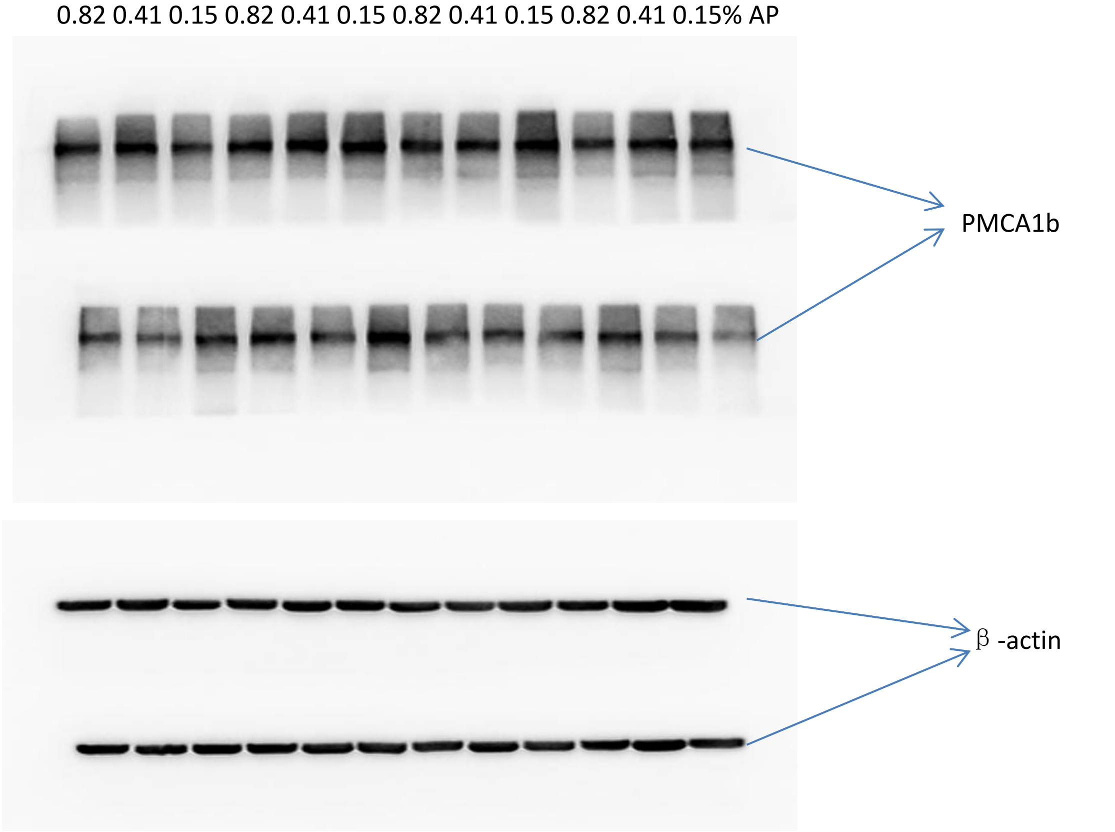

Fig S9 Effects of dietary available phosphorus levels protein expression level of CaBP-D28k in jejunum (Fig 3H)

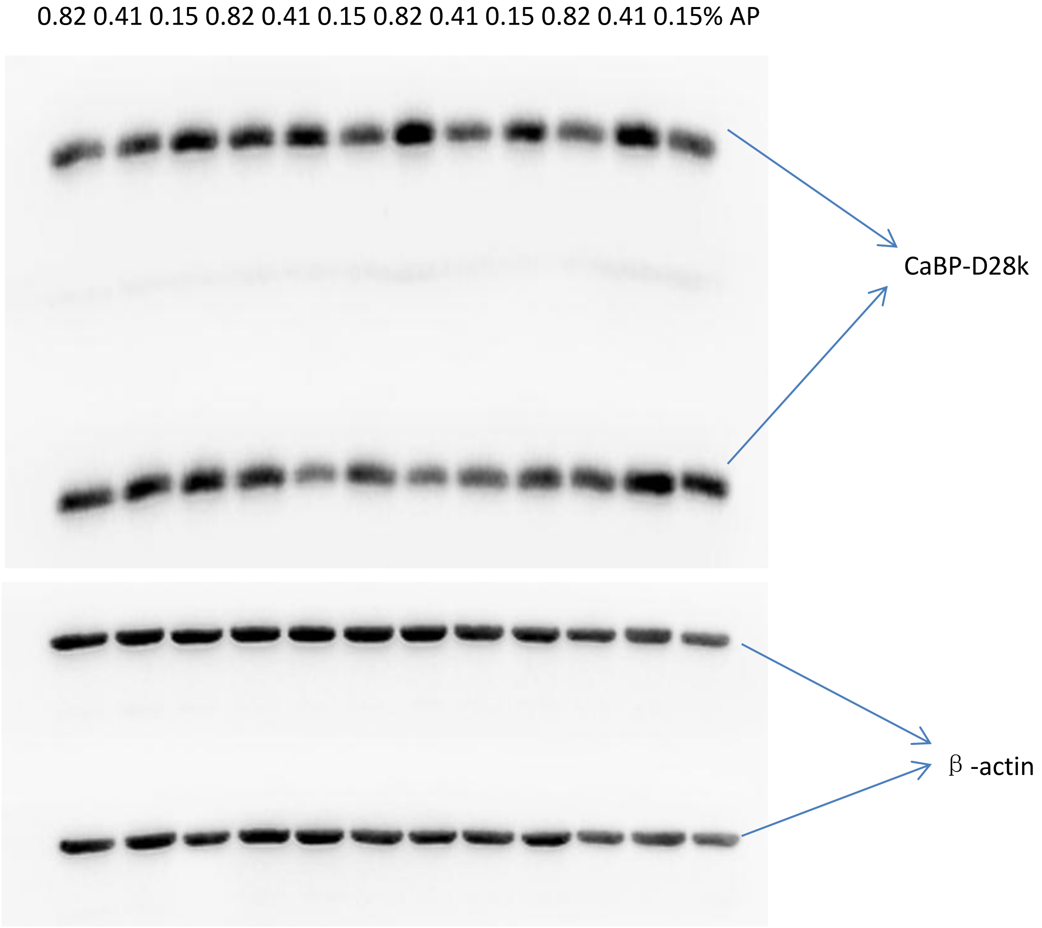

Fig S10 Effects of dietary available phosphorus levels protein expression level of PMCA1b in jejunum (Fig 3K)

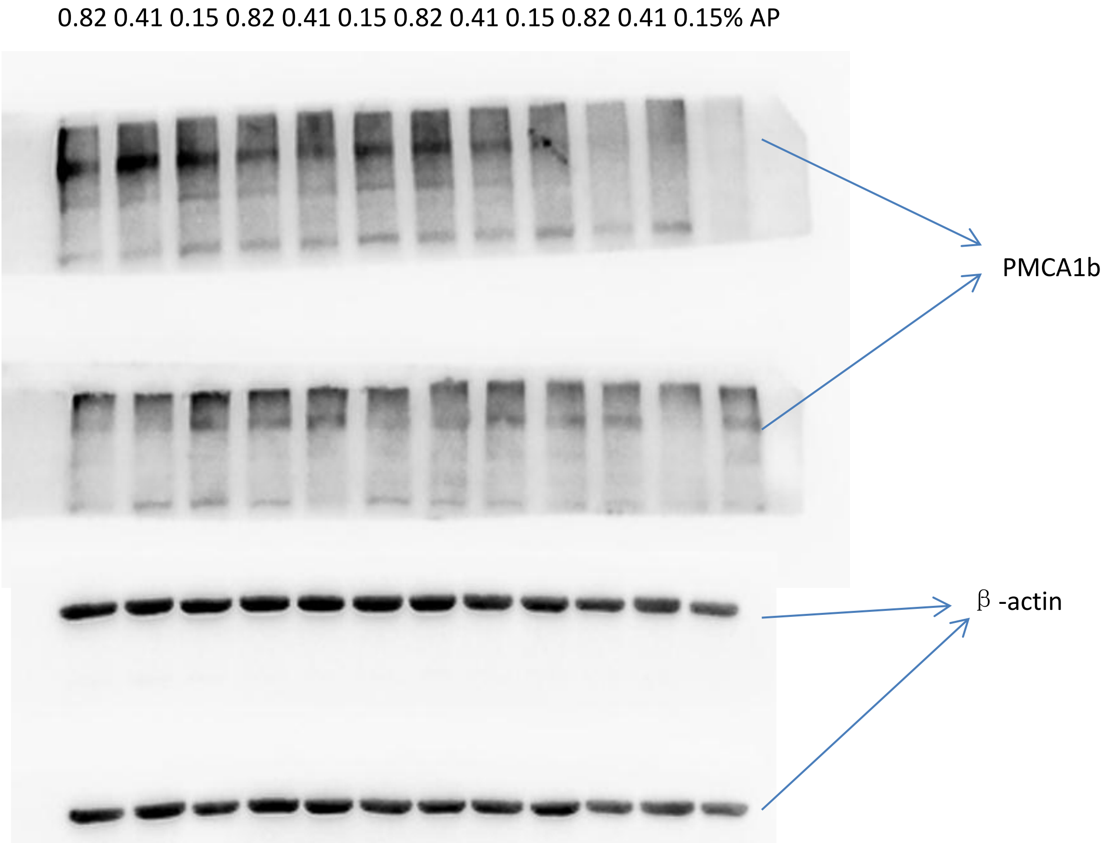

Fig S11 Effects of dietary available phosphorus levels protein expression level of CaBP-D28k in ileum (Fig 3N)

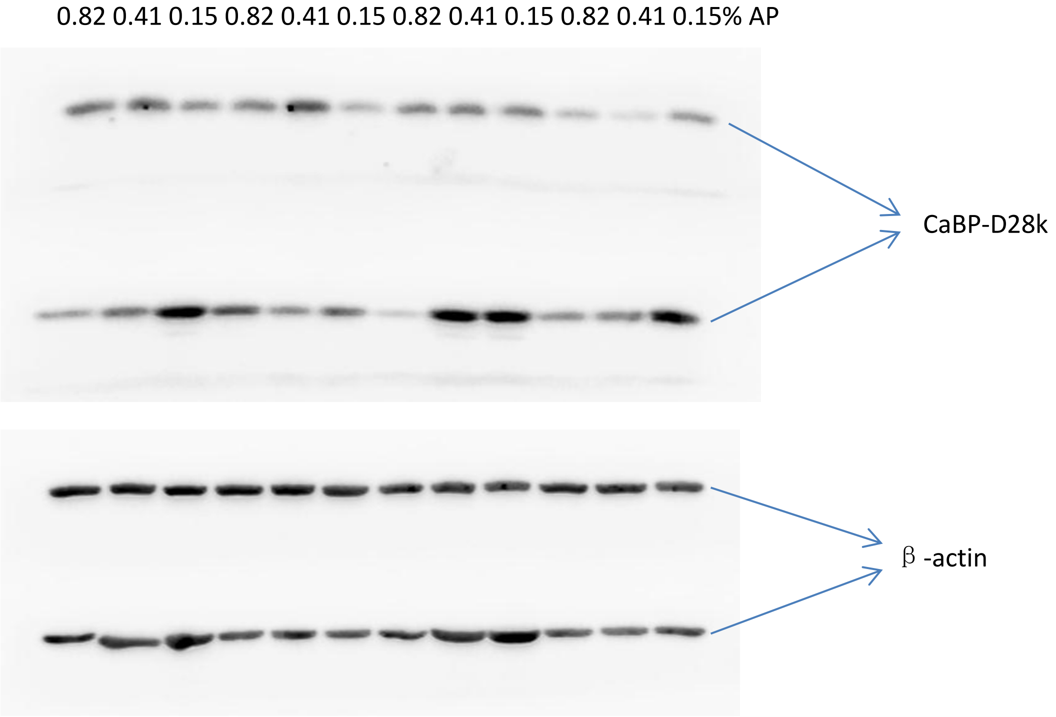

Fig S12 Effects of dietary available phosphorus levels protein expression level of PMCA1b in ileum (Fig 3N)

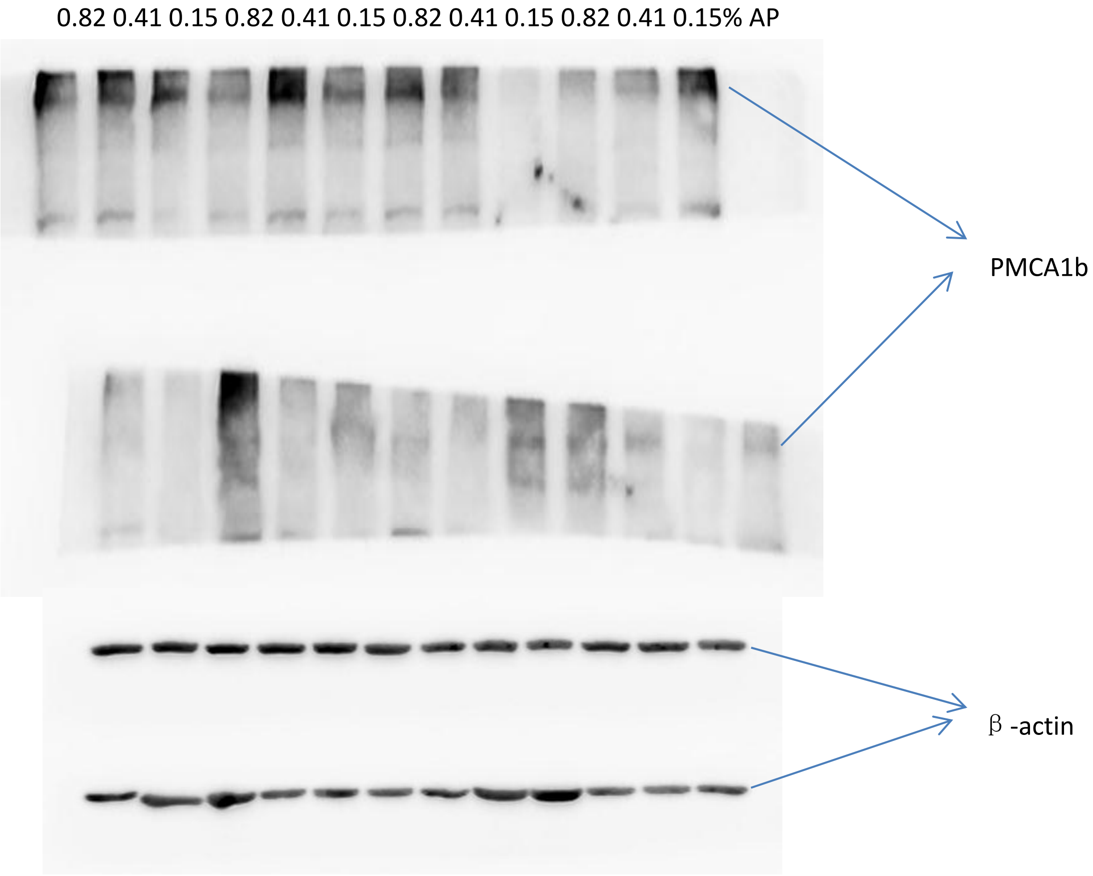

Fig S13 Effect of dietary available phosphorus levels on the protein expression level of CaBP-D28k in kidney (Fig 4C)

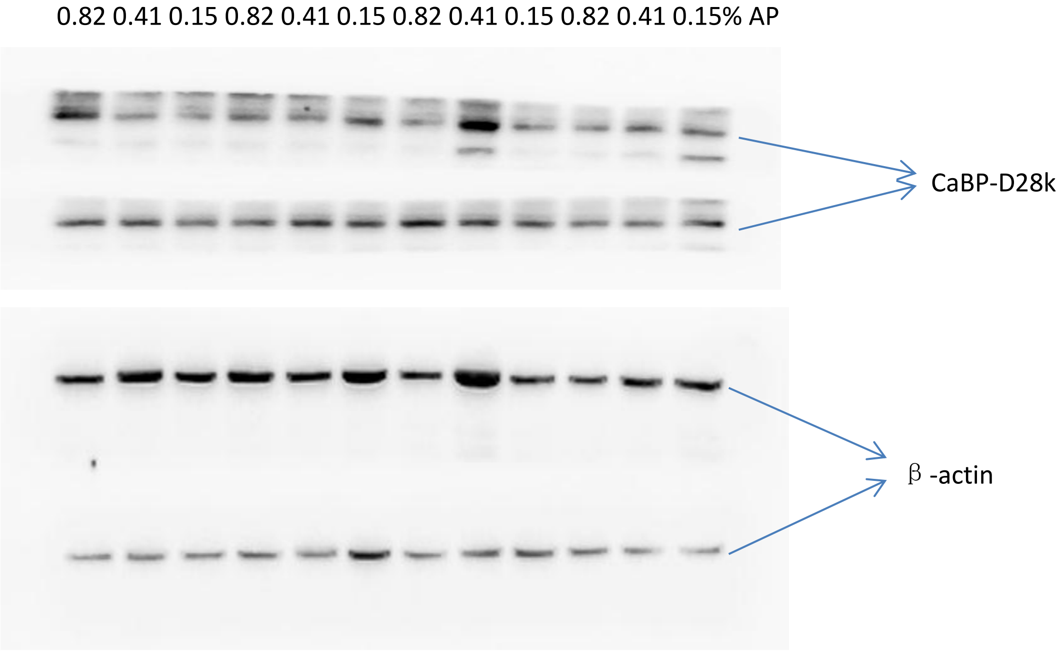

Fig S14 Effect of dietary available phosphorus levels on the protein expression level of PMCA1b in kidney (Fig 4F)

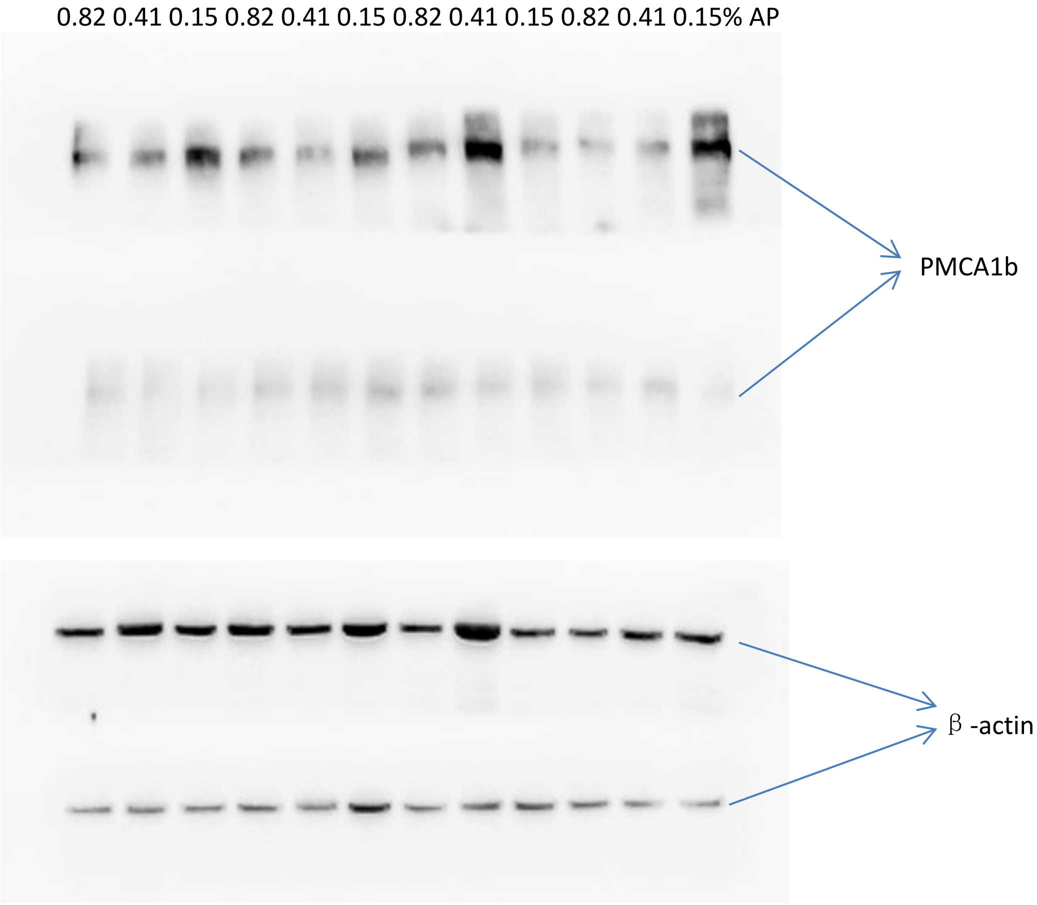

Fig S15 Effect of dietary available phosphorus levels protein expression level of CaBP-D28k in shell gland (Fig 5B)

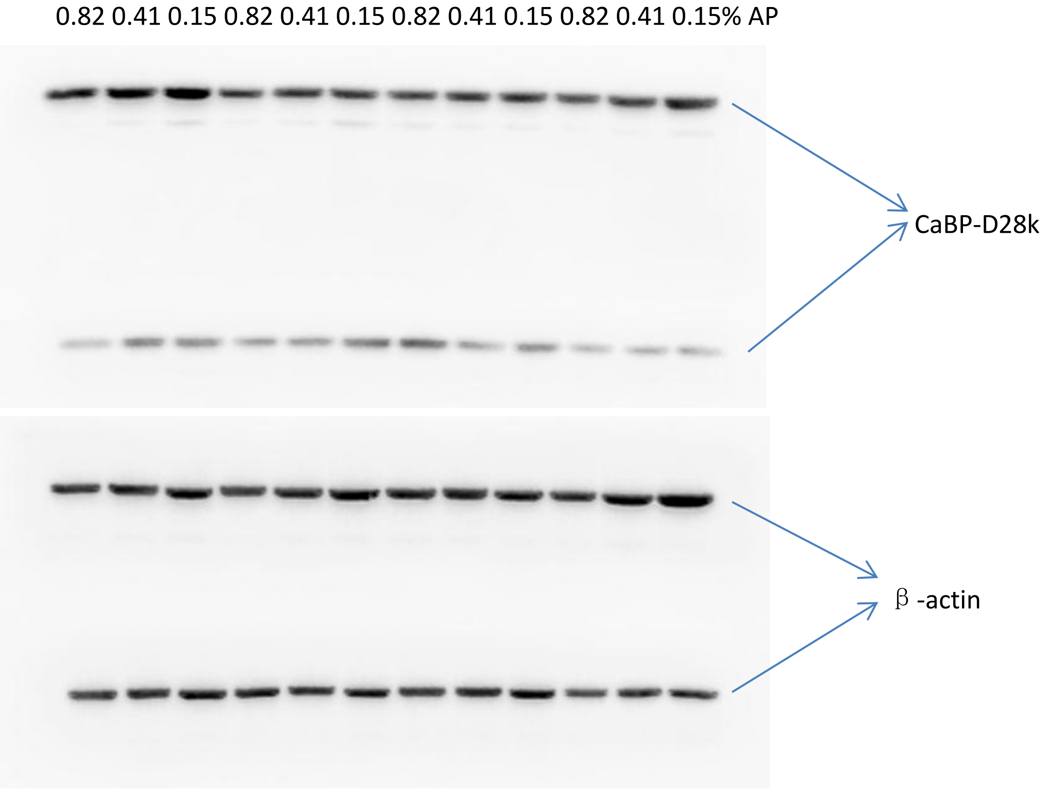

Fig S16 Effect of dietary available phosphorus levels protein expression level of PMCA1b in shell gland (Fig 5E)

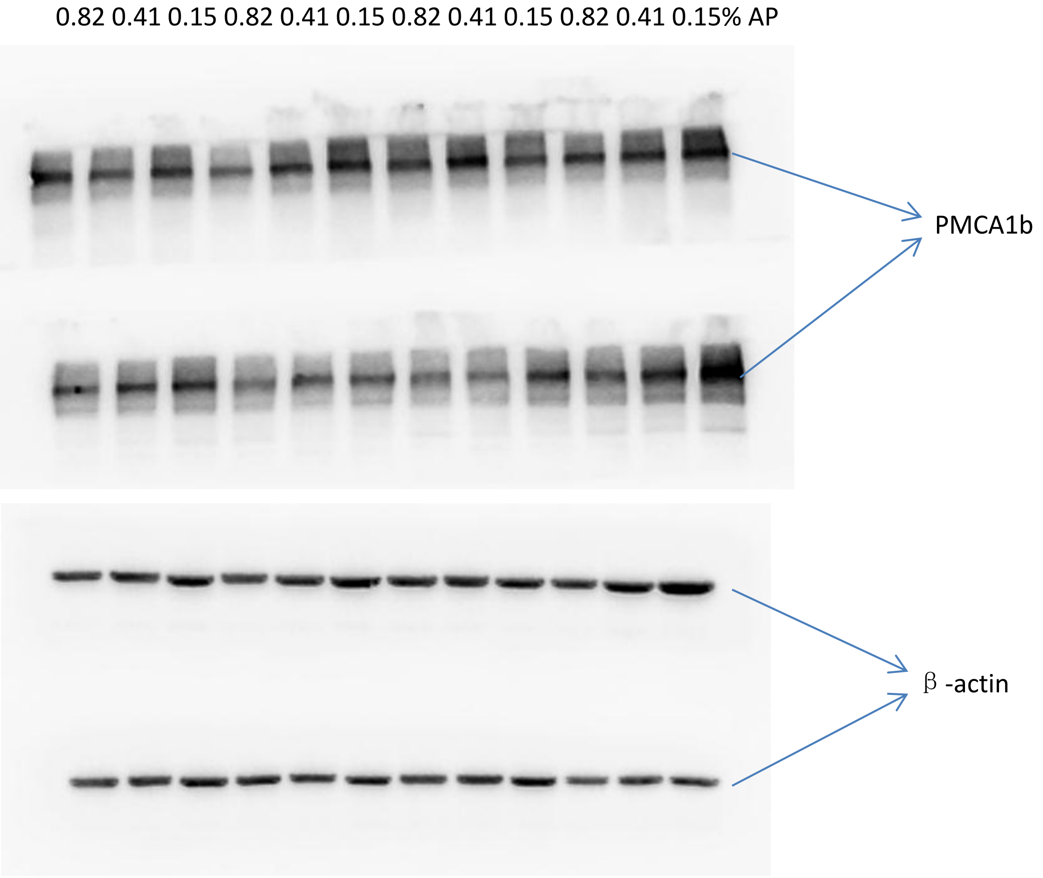

Supplement: Supplementary file 1 [file Table_1.pdf]
